# A Meta-Analysis Indicates Positive Correlation between Genetic Diversity and Species Diversity

**DOI:** 10.3390/biology10111089

**Published:** 2021-10-23

**Authors:** Lei Xie, Yuan Yang, Yao Li, Shuifei Chen, Yueyao Feng, Ningjie Wang, Ting Lv, Hui Ding, Lu Wang, Yanming Fang

**Affiliations:** 1 Co-Innovation Center for Sustainable Forestry in Southern China, Key Laboratory of State Forestry and Grassland Administration on Subtropical Forest Biodiversity Conservation, College of Biology and the Environment, Nanjing Forestry University, Nanjing 210037, China; nlxielei@njfu.edu.cn (L.X.); Yangyuan@njfu.edu.cn (Y.Y.); liyaolisantu@njfu.edu.cn (Y.L.); fyy1998@njfu.edu.cn (Y.F.); wnj@njfu.edu.cn (N.W.); lvtingnanlin@njfu.edu.cn (T.L.); wanglunjfu@gmail.com (L.W.); 2 Research Center for Nature Conservation and Biodiversity, State Environmental Protection Scientific Observation and Research Station for Ecology and Environment of Wuyi Mountains, State Environmental Protection Key Laboratory on Biosafety, Nanjing Institute of Environmental Sciences, Ministry of Ecology and Environment, Nanjing 210042, China; chenshuifei@163.com (S.C.); dinghui@nies.org (H.D.)

**Keywords:** biodiversity, community genetic, meta-analysis, genetic differentiation, community dissimilarity, SGDC

## Abstract

**Simple Summary:**

Understanding species and genetic correlations (SGDCs) is essential to establish community composition. In this study, 295 observations from 39 studies explored the SGDCs and the underlying drivers through conducting a global meta-analysis. A positive correlation was found, suggesting that parallel processes (environmental heterogeneity, area, and connectivity etc.) have effects on two diversities. As current biodiversity hotspots have mainly been identified based on high species diversity and high endemism of taxon, the understanding of SGDC will substantially help us to determine whether and how genetic diversity can be used in identifying biodiversity hotspots, as well as in developing conservation practices and policies for biodiversity.

**Abstract:**

Species diversity (SD) and genetic diversity (GD) are the two basic levels of biodiversity. In general, according to the consensus view, the parallel effects of environmental heterogeneity, area, and connectivity on two levels, can drive a positive correlation between GD and SD. Conversely, a negative correlation or no correlation would be expected if these effects are not parallel. Our understanding of the relationships between SD and GD among different ecosystems, sampling methods, species, and under climate change remains incomplete. In the present study, we conducted a hierarchical meta-analysis based on 295 observations from 39 studies and found a positive correlation between genetic diversity and species diversity (95% confidence interval, 7.6–22.64%). However, significant relationships were not found in some ecosystems when we conducted species–genetic diversity correlation analysis based on a single ecosystem. Moreover, the magnitudes of the correlations generally decreased with the number of sampling units and the annual average the temperature of sampling units. Our results highlight the positive correlation between GD and SD, thereby indicating that protecting SD involves protecting GD in conservation practice. Furthermore, our results also suggest that global increases in temperature during the 21st century will have significant impacts on global biodiversity.

## 1. Introduction

The current ongoing loss of biodiversity is far greater than that at any time in human history [1]. Increases in global atmospheric CO_2_, drought events, land-use intensity, and climate change are major challenges that affect the maintenance of biodiversity and other ecosystem functions [2,3,4]. The conservation of biodiversity is essential for the sustainable development of mankind. Species diversity (SD) and genetic diversity (GD) are the two basic levels of biodiversity [5,6,7,8]. Understanding the variations in SD in time and space is a central goal of community ecology [9]. Exploring the intraspecific genetic structure and related factors is a core target of population genetics [10]. However, studies of community compositions and intraspecific genetic structure have generally been conducted separately by investigators from different disciplines, and thus there has been little progress in understanding the relationships between GD and SD for many years [11,12,13]. Fundamentally, species–genetic diversity correlation (SGDC) analysis can be applied to clarify the interactions between intraspecific variation and the community composition at various trophic levels [14]. If this relationship holds, we can predict GD through SD or predict SD through GD at a certain scale, which is important for biodiversity conservation and social production in practice [15].

In previous studies, the community assembly was ignored when studying intraspecific genetic structures [16]. Thus, GD has not been considered a high priority for conservation based on the long-term persistence of species [17]. It is not surprising that the GD of focal species are neglected when studying SD. In short, we know little about the relationship between SD and GD. Therefore, elucidating the relationship between SD and GD is critical for evaluating the impacts of the degree of association between SD and GD caused by changes in environmental heterogeneity on the rapid decline in biodiversity [18,19].

In community ecology, the niche hypothesis suggests that SD is negatively correlated with GD because high SD should constrain the GD within species [14]. However, Vellend [20] proposed that community assembly involves four basic processes comprising drift, selection, dispersal, and speciation, which are similar to the four processes in population genetics. In other words, the forces that lead to population and community changes seem to be the same [21]. Therefore, we expect that there should be a positive correlation between SD and GD [22] due to the parallel effects of environmental heterogeneity on SD and GD. However, Lamy et al. [23] found that significant negative SGDCs are as frequent as positive SGDCs. In their view, ‘site factors’ such as environment condition, area and connectivity, and ‘community factors’ were two types of factors affecting SGDCs.

Some recent experiments have explored the relationships between SD and GD in plants and animals among different ecosystems, where a common local species was usually selected as a focal species. Genetic diversity such as the allelic richness and expected heterozygosity can be measured by molecular markers. In addition, the species diversity such species richness and evenness at the location of the focal species can be surveyed through observation plots. The significance of the correlation between SD and GD can be detected by measuring the two indicators. Considering the temporal and spatial changes in the two indicators, two experiments should be conducted at the same time in one place. For example, Vellend [24] selected *Trillium grandiflorum* as a focal species and found that both the SD and GD were lower in secondary forests compared with primary forests, thereby implying that SGDC was impacted by human interference. Interestingly, both the SD and GD can be affected in parallel by land use, and the land-use intensity was the main driving force that affected the positive SGDC. By contrast, the different responses of SD and GD to environmental heterogeneity might contribute to negative and zero SGDCs. For instance, it was shown that the SD was affected by the biological characteristics in dry grassland, whereas the GD mainly responded to the fragmented geography. Thus, the incongruent effects of environmental heterogeneity on two levels caused a mismatch in SD and GD [25]. In alpine plant communities, a large-scale study found no correlation between SD and GD due to the effects of environmental and glacial refugia on GD [15]. Moreover, environmental heterogeneity in terms of parameters, such as soil nutrition and topography, might promote species coexistence, thereby constraining the growth of the focal species and reducing its effective population size, and thus the correlation observed between the GD and SD would be negative [26]. In a well-connected area, the relationship between SD and GD may indicate whether there is a conflict between conserving two levels of biodiversity. For instance, a positive correlation suggests that protecting SD is equivalent to protecting GD, whereas a negative correlation might indicate that there is a conflict between protecting GD and protecting SD [27].

A previous meta-analysis specifically investigated the relationship between plant SD and GD [28] and obtained contrasting conclusions, i.e., the relationship between adaptive GD and SD was positive but small, and no correlation was found with the neutral GD and SD. This study only examined the response of adaptive or neutral GD to SD, but it did not determine the effects of climate changes, the sampling units, and other factors on the SGDC. Vellend et al. [29] preferred to refer to their method as data integration analysis rather than meta-analysis, but they determined none of the results obtained by meta-analysis, such as the effect size, sampling variance, and cumulative effect size. Moreover, the probability of type I error increased because they failed to consider the autocorrelations among observations [29,30]. In addition, the time between some cases exceeded ten years in several experiments and they did not consider the changes in community assembly and population structure, and thus there may be doubt regarding whether they were actually related because there must be differences in the species diversity and genetic diversity at different ages [22,31]. Some recent experiments were specifically conducted based on the SGDC, and thus it is possible to synthesize the results of these experiments to explore the SGDC on a global scale.

**Figure 1:**
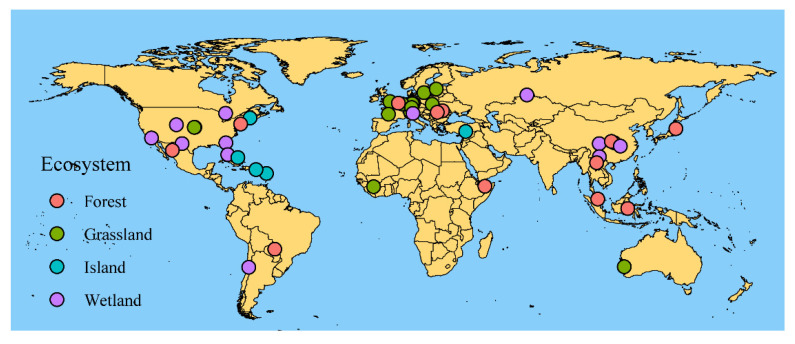
Geographic locations of the 39 studies included in the meta-analysis. Different colors indicate different ecosystem types.

In the present study, we performed a global meta-analysis based on 295 observations from 39 studies (Figure 1, Table S1) in order to test the correlations between SD and GD. This meta-analysis based on substantially expanded data sets allowed us to examine multiple ecosystems simultaneously and in depth. The questions addressed in this study are as follows. (1) Is the GD in one or more focal species congruent with the species diversity in the community assembly? (2) Does SGDC differ among various ecosystems, species, sampling units, and molecular markers? (3) Do the number of sampling units and climate factors significantly affect the SGDC? The answers to these questions are important for understanding and modeling the relationships between SD and GD.

## 2. Materials and Methods

### 2.1. Data Collection

The studies considered were retrieved using Web of Science, Science Direct, and the China Nation Knowledge Infrastructure (CNKI) up to June 2021 (Figure S1). Different keywords and combinations were used, such as “correlation between species diversity and genetic diversity”, “SGDC”, and “community assembly and genetic structure”. Some of the data used by Vellend et al. [29], who proposed the theory of SGDC, were also employed in the present study. However, only 7 studies with 9 observations considering the varieties of species diversity and genetic diversity were included. The following criteria were applied in this study. (1) We preferred to select studies that conducted experiments at two diversity levels in one place at the same time. (2) Data for SD and GD were required. (3) Measurements of two levels must have been obtained under the same biotic conditions in the field. (4) The correlation coefficients, *p*-values, and the number of sample units must be reported, or they could be extracted from the publications. (5) Some studies reported two levels measured at different times in the same place and we included studies with a time difference provided that the time did not exceed five years considering the changes in the community and genetic structure.

Whenever possible, we extracted the number of sample units, whether the sampling unit was discrete, as well as the type of ecosystem, correlation coefficient between the SD and GD, species, and molecular marker, if reported. If the author did not calculate the correlation coefficient between SD and GD in the original text, we extracted this part of the information using WebPlotDigitizer [32], calculated the correlation coefficient, and tested whether the correlation between the two levels was significant. We extracted the coordinates (WGS84) of the study sites based on their descriptions in the studies. The mean annual precipitation (MAP) (mm) and mean annual temperature (MAT) (°C) were recorded from studies, or derived from the WorldClim (http://www.wordclim.org/, accessed on 1 June 2021) database using latitude and longitude. To the best of our knowledge, this is the first meta-analysis to investigate the correlations between SD and GD in different ecosystems and species pools. We also explored the effects of various geographical types, molecular markers, and the number of sampling units on SGDC.

Our meta-data set contained correlation coefficients from −0.94 to 0.98 and the number of the sampling units ranged and from 4 to 137. Most of the ecosystem types comprised forests, islands, grasslands, and wetlands. Forests, wetlands, and grasslands accounted for 49.13%, 25.09%, and 16.38% of the data set, respectively, based on 295 observations from 53 focal species in 39 published papers (Table S1). To explore the effects of different species pools and genetic methods on SGDCs, categorical moderators, such as species pools and molecular markers, were also included.

### 2.2. Statistical Analyses

To control for non-independence in the data due to multiple effect sizes per study and focal species, we performed all analyses in R (4.1.1), running a hierarchical meta-analysis by the “metafor” package [33], with the restricted maximum likelihood (REML) method. The species nested in reference was defined as a random factor, using function ‘rma.mv’. Statistical correlation coefficients were employed to determine the effect size with Fisher’s *Z*. Fisher’s *Z* was calculated using the following formula where *r* is the correlation coefficient and *Zr* is the effect size.

(1) $$Zr=\frac{1}{2}\ln(\frac{1+r}{1-r})$$

Fisher’s *Z* > 0 or Fisher’s *Z* < 0 indicates whether there are positive or negative correlations between SD and GD, respectively. The sampling variance (vi) associated with each Fisher’s *Z* was determined using the following formula where *n* represents the sample size for *r*.

(2) $$\overset{\wedge }{\partial }{Zr}^{2}=\frac{1}{n-3}$$

Furthermore, based on mixed effects models, the cumulative effect size was calculated to assess whether there was a correlation between SD and GD. The cumulative effect size was calculated as follows:

(3) $$\bar{y}=\frac{\sum_{i=1}^{k}w_{i}^{*}y_{i}}{\sum_{i=1}^{k}w_{i}^{*}}$$

where $w_{i}^{*}=1/(vi+\tau^{2})$ (*i* = 1, 2, 3, …, *k*) is the weight for one study and $y_{i}$ is the effect size.

Similar to general biological meta-analysis, significant residual heterogeneity was found by random-effects meta-analysis of the data set (Qt = 5090.6912, *p* < 0.0001), which we tried to explain with different variables. In particular, we considered categorical variables (ecosystem types, species pool etc.) and continuous variables (MAT, MAP, number of sampling units) to explain their influence on the effect size.

Finally, a funnel plot was prepared and sensitivity analyses were conducted to test for publication bias (Figure S3).

## 3. Results

**Figure 2:**
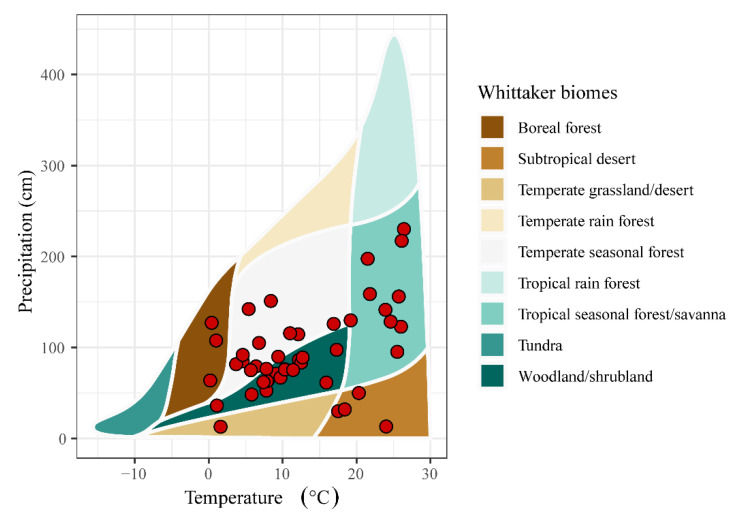
Environmental locations of the 39 studies across Whittaker’s biomes.

After document retrieval and full text screening, 39 studies and 295 observations were retained for our meta-analysis (Figure 1, Table S1), where 39 study sites spanned 94.46° latitude (from 64.46° N to 30° S) and. 278.87° longitude (from 140.69° E to 138.18° W). Our data set covered six Whittaker’s biomes, excluding Antarctica, and SGDC values were conducted for these studies (Figure 2).

### 3.1. Evaluation of Total Heterogeneity

Across all the selected studies, we found a positive correlation between genetic diversity and species diversity (estimate = 0.1512 *p* < 0.001; Figure S2). A positive cumulative size effect was found based on the random-effects model using REML (95% confidence interval (CI), 7.6–22.64%). Thus, on average, there was a positive correlation between SD and GD; however, the correlation is weak. Moreover, we determined the two sides of the funnel graph to be roughly symmetrical (Figure S3).

**Figure 3:**
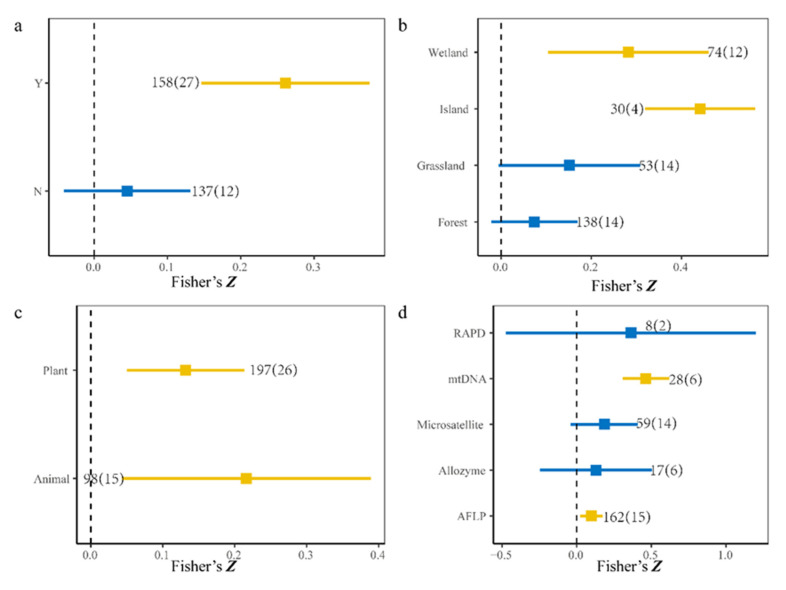
Cumulative effect sizes among different groups. The numbers of observations and studies are shown outside and inside the parentheses, respectively. (**a**) Y denotes discrete sampling and N denotes continuous sampling. (**b**) Cumulative effect sizes among different ecosystem types. (**c**) Cumulative effect sizes using different species. (**d**) Cumulative effect sizes using different molecular markers. Blue and yellow quadrates represent variables with 95% confidence intervals that do not overlap zero (*p* < 0.05), and overlap zero (*p* > 0.05), respectively.

Furthermore, after separate analyses, the 295 observations were divided into two, four, two, and five groups based on the sampling methods, ecosystem types, species pool, and molecular marker, respectively. We found that the sampling units were discrete and a positive relationship was determined, but no relationships between SD and GD were detected for continuous sampling units (Y: estimate = 0.26, *p* < 0.01, 95% CI, 14.63–37.58%; N: estimate = 0.05, *p* > 0.05, 95% CI, −4.09% to 13.10%, Figure 3a). Furthermore, regardless of whether the focal species was an animal or a plant, the correlation between SD and GD was positive (animals: estimate = 0.22, *p* = 0.01, 95% CI, 4.3–38.93%; plants: estimate = 0.13, *p* < 0.01, 95% CI, 5.00–21.24%, Figure 3c). However, early molecular marker methods such as allozymes and RAPD (random amplified polymorphic DNA) might have caused a mismatch in SGDC (amplified fragment length polymorphisms (AFLP): estimate = 0.10, *p* < 0.01, 95% CI, 2.31–17.43%; allozymes: estimate = 0.13, *p* > 0.05, 95% CI, −24.58% to 50.44%; microsatellites: estimate = 0.18, *p* > 0.05, 95% CI, −4.09–41.05%; mtDNA: estimate = 0.46, *p* < 0.01, 95% CI, 30.77–61.89%; RAPD estimate = 0.36, *p* > 0.05, 95% CI, −47.39–1.2%, Figure 2d). We also found significant correlations in ecosystems (wetland: estimate = 0.28, *p* < 0.01, 95% CI, 10.38–46.04%; island: estimate = 0.44, *p* < 0.01, 95% CI, 31.88% to 56.33%, Figure 3b). On the contrary, we did not find a significant positive correlation between SD and GD in forest and grassland (grassland: estimate = 0.15, *p* > 0.05, 95% CI, −0.5–30.85%; Forest: estimate = 0.07, *p* > 0.05, 95% CI, −2.21% to 16.94%, Figure 3b).

### 3.2. Factors That Affected SGDC

To explain the significant residual heterogeneity, we determined the influence of categorical variables and continuous variables on the effect size. In this part, we found that effects of mean annual temperature (MAT) and mean annual precipitation (MAP) on Fisher’ *Z* of SGDC were inconsistent.

**Figure 4:**
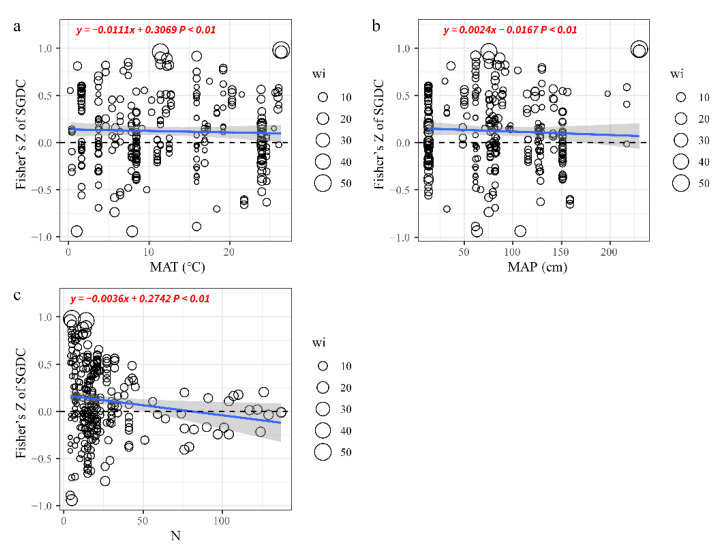
Effects of continuous variables on SGDC. (**a**) MAT: mean annual temperature, (**b**) MAP: mean annual precipitation, (**c**) N: number of sampling units. Partial linear regressions were used to fit the blue lines and the 95% CIs are presented as shaded areas. The significance (*p*-value) is shown for the tested relationship. The sizes of the circles represent the weights of the observations.

The number of sampling units and MAT had significant negative effects on SGDC (Figure 4a,b), with Qm values of 34.78 and 17.07, respectively. The effect size of SGDC decreased as the number of sampling units and MAT increased. It is worth noting that with the increase of sampling units, it may cause the SGDC to show a negative value (Figure 4c). However, the effect size of SGDC increased as MAP increased with Qm values of 15.97 (Figure 4b). The categorical variables comprising discrete sampling units, ecosystem, species pools, and molecular markers were employed to explain the residual heterogeneity. The responses of SGDC to the discrete sampling method and species pools were significant and positive (Table 1). In addition, the choice of focal species might have influenced the SGDC results. However, the effects of ecosystems and molecular markers on SGDC were inconsistent, e.g., allozyme, RAPD, grassland, and forest had no influence on SGDC (Table 1).

We also explored the impact of the interaction between categorical variables on SGDC. It is worth noting that the interaction between discrete sampling methods and most categorical variables has a positive effect on SGDC (Table S2). This showed that the sampling strategy is crucial for exploring the relationship between SD and GD.

## 4. Discussion

Our findings provide new insights into the exploration of SGDC at different scales, as well as showing that some types of ecosystems have not been found to have positive correlation between genetic diversity (SD) and species diversity (GD). For this reason, we need to explore environmental properties on genetic diversity and species diversity in different ecosystems, especially in grassland and forest. First, in general, a positive SGDC was found in this meta-analysis (Figure S1). Second, we found that SGDC decreased as the number of sampling units and MAT increased; however, MAP decreased (Figure 4). Third, a wide range of geographic variations, ecosystems, and sampling methods might result in non-significant SGDC values, but the selection of focal species and molecular marker methods used for measuring genetic structure affected the SGDC (Table 1, Table S2).

### 4.1. Positive Correlation between SD and GD

We determined a significant positive relationship between SD and GD, which is consistent with previous findings [28,29] (Figure S1) that environment heterogeneity has parallel effects on SD and GD. In addition, our results suggest that two neutral ecological processes comprising drift and dispersal mainly affect the changes in the community composition [34]. This is likely because competition, the carrying capacity within a community, and habitat connectivity among sites will change the structures of the community and population through ecological process, such as drift and dispersal [35].

Our analyses of the effect sizes for different components showed that the SGDC values were not significant in ecosystems such as grasslands, forests (Figure 3b), which is consistent with previous studies [25,36], and thus the SD and GD respond differently to environment heterogeneity. To the best of our knowledge, most previous studies in grasslands investigated continuous sampling units at the same site [37,38], which may also be an important reason why positive correlations were not observed. In addition, the life cycle in grassland could have an important effect when monitoring genetic differentiation [39,40]. Unexpectedly, a further novel finding is that our meta-analysis showed, on average, a positive correlation between SD and GD in forest ecosystem, but not significant (Figure 3b). The intricate interaction between biological and non-biological factors may be the main reason for the mismatch between SD and GD. Forest is one of the most important ecosystems in the terrestrial ecosystem, but land use [24], resource competition [26], and latitudinal biodiversity gradient (LBG) [36] would have varying degrees of impact on the community assembly and population structure in the forest ecosystem.

### 4.2. Effects of Species and Experimental Methods on SGDC

The methods used for testing GD appeared to have positive effects on SGDC (Table 1). Most of the methods that are currently used for detecting genetic structure are biased toward simple sequence repeats, AFLPs, and single nucleotide polymorphisms [36,41,42]. Thus, the methods used for detecting genetic structure did not hinder the determination of SGDC. Although neutral markers such as SSR cannot reflect the relationship between species and ecological selection, they can all reflect population drift, selection, etc., and indirectly affect community assembly by affecting population structure [23]. It should be noted that in neutral theory, drift and migration are the causes of changes in species and community structures, and they are largely affected on two levels [20]. If SD or GD are affected by these two ecological processes, then it has been suggested that a positive SGDC is a consequence according to neural theory [20].

Similar to general empirical research results, the SGDC values were positive in animals and plants (Figure 3), and thus the focal species selected did not have significant effects on the relationships between SD and GD regardless of whether they studied the variation in animals or plant community assemblies and genetic structure. In addition, both the animals and plants selected had positive effects on SGDC (Table 1), which suggests no strong relationship between the focal species selected and SGDC. Interesting, we found that the effects of animals on SGDC were greater than those of plants, possibly because plants have a lower capacity for dispersal than animals [43,44]. Plants can only move their alleles between populations in the form of seeds and pollen, whereas animals have greater mobility, which facilitates the exchange of their alleles to increase GD.

### 4.3. Effects of Climate Change and Numbers of Sampling Units on SGDC

Despite the wide range of ecosystem types considered, including wetland, island grassland, and forest, the responses of SGDC to MAT and the number of sampling units (N) were consistent. As MAT and N increased, the SGDC values became weaker (Figure 4). The results also suggested that climate change and the sampling area had great effects on SGDC. Similar to the findings reported by Taberlet et al. [15], the sampling area had to increase as N increased, thereby causing spatial variations in SD and GD [29,43]. In addition, according to theories of environmental filtering, the changes in SD along an environmental gradient can be interpreted as the environment filtering out of species that are not suitable for the local environmental conditions [45,46]. This is the main reason why increases in the number of N led to decreases in both SD and GD, thereby making the correlations weaker or even zero.

Our results also revealed the impact of global climate change on biodiversity. We found that an increase in MAP can increase the positive correlation between SD and GD, while MAT does the opposite (Figure 4b,c). It is generally accepted now that in the context of global climate change, changes in water, heat, and other conditions would inevitably break the interrelationships between animal and plant species in the natural ecosystem after long-term adaptation and evolution [47], and further lead to changes in the biodiversity pattern in the ecosystem, which in turn will cause changes in the structure and function of the ecosystem [48].

### 4.4. Implications for Future Experimental Design Regarding SGDC

Many previous studies ignored the impact of environmental heterogeneity on SGDC and explored the relationships between genetic differentiation and community dissimilarity (β-SGDC) [49,50]. Thus, in the next step, we should consider is how to detect SGDC along an environmental gradient. The relationships between “distance-decay” pattern among community assembly and the “isolation by distance” pattern among the population should be considered in the SGDC, which would help us better understand the role of migration and connectivity in SGDC [23]. Here, we used the data set of Lamy et al., Watanabe et al., and Pfeiffer et al. to carry out a simple meta-analysis of β-SGDC, following the methods of these articles. We found a more positive correlation among β-SGDC (estimate = 0.2544, *p* < 0.001; Figure S4). Therefore, figuring out relationships between genetic differentiation and community dissimilarity was helpful to better establish community assembly.

Clearly, in large-scale laboratories, selecting molecular marker methods should not be a problem when we need to consider economic benefits. However, our understanding of the extent to which environmental heterogeneity affects SD and GD is still lacking. In fact, both topography and soil factors have major effects on the community assembly [26]. The lack of explanatory variables such as soil nutrient elements and topography prevented us from identifying the sources of most of the variation in the effect size [51]. It is clear that both genetic structure and community composition are affected by environmental heterogeneity. However, whether communities and populations would affect each other and further affect SGDC remains to be scientifically proven. Therefore, in our meta-analysis, we calculated the effect size based on the correlation coefficient, and thus we neglected the impacts of SD and GD themselves on SGDC.

## 5. Conclusions

In this study, we found an overall positive correlation between genetic diversity and species diversity. However, the correlations between SD and GD were not significant in some ecosystems such as forests and grasslands when analyzed separately (Figure 3, Figure S2). These relationship between SD and GD were less significant or even negatively correlated as the number of sampling units and MAT increased (Figure 4a,c). However, there was a positive relationship between MAP and effect size of SGDC (Figure 4b). Furthermore, different ecosystems could affect the SGDC results (Table 1). Our results suggest that protecting SD will also protect GD on a certain scale. Our findings have significant implications for elucidating species coexistence and the maintenance of biodiversity in community assembly studies. From a long-term perspective, studying ecological communities and population genetics separately is outdated, and it is necessary to consider both levels at the same time as well as to determine the underlying associated mechanisms [11,34]. Our results also suggest that global increases in temperature during the 21st century will have great impacts on global biodiversity.

## Figures and Tables

**Table 1:** Effects of categorical variables (sampling methods, ecosystems, species pools, and molecular markers) on effect size of SGDC.

Types	Attribute	Estimate	SE	*p*-Value
Sampling methods	Discrete	0.25	0.05	<0.01
	Continuous	0.05	0.05	0.39
Ecosystems	Forest	0.08	0.05	0.12
	Grassland	0.13	0.09	0.12
	Island	0.45	0.17	<0.01
	Wetland	0.28	0.08	<0.01
Species pools	Animal	0.21	0.07	<0.01
	Plant	0.13	0.04	<0.01
Molecular markers	AFLP	0.10	0.04	0.03
	Allozyme	0.11	0.12	0.33
	Microsatellite	0.26	0.08	<0.01
	mtDNA	0.33	0.10	<0.01
	RAPD	0.28	0.26	0.28
	SNP	0.07	0.28	0.18

## Data Availability

The datasets and R code used and/or analyzed during the current study are available from the Figshare https://figshare.com/articles/dataset/Meta_SGDC/16635376.

## Supplementary Materials

The following are available online at https://www.mdpi.com/article/10.3390/biology10111089/s1, Figure S1: Workflow diagram showing the procedure for selecting publications; Figure S2: The 295 effect sizes estimate for SGDC; Figure S3: Funnel plot used to determine publication bias; Figure S4: The 61 effect sizes estimate for β-SGDC; Table S1: Information for 40 studies; Table S2 Interaction of two categorical variables on effects of SGDC, *p*-value < 0.05 are shown in the table.
