# Supplementary material for: A Meta-Analysis Indicates Positive Correlation between Genetic Diversity and Species Diversity
Source: Biology (Basel). 2021 Oct 23;10(11):1089. doi: 10.3390/biology10111089 (PMC8615265; doi:10.3390/biology10111089)
Supplement: Supplementary file 1 [file biology-10-01089-s001.zip › biology-1405656-supplementary.pdf]

Supplementary meta-analysis

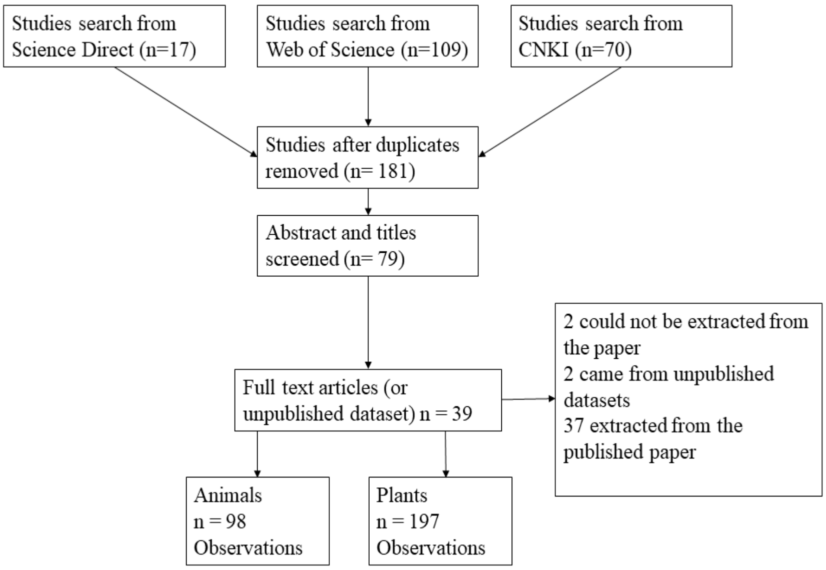

Figure S1. Workflow diagram showing the procedure for selecting publications.

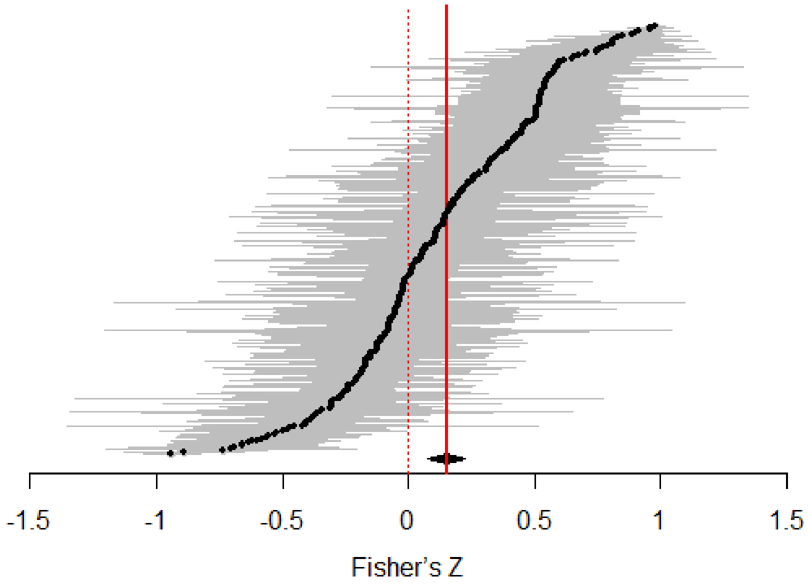

Figure. S2. The 295 effect sizes estimate for SGDC.

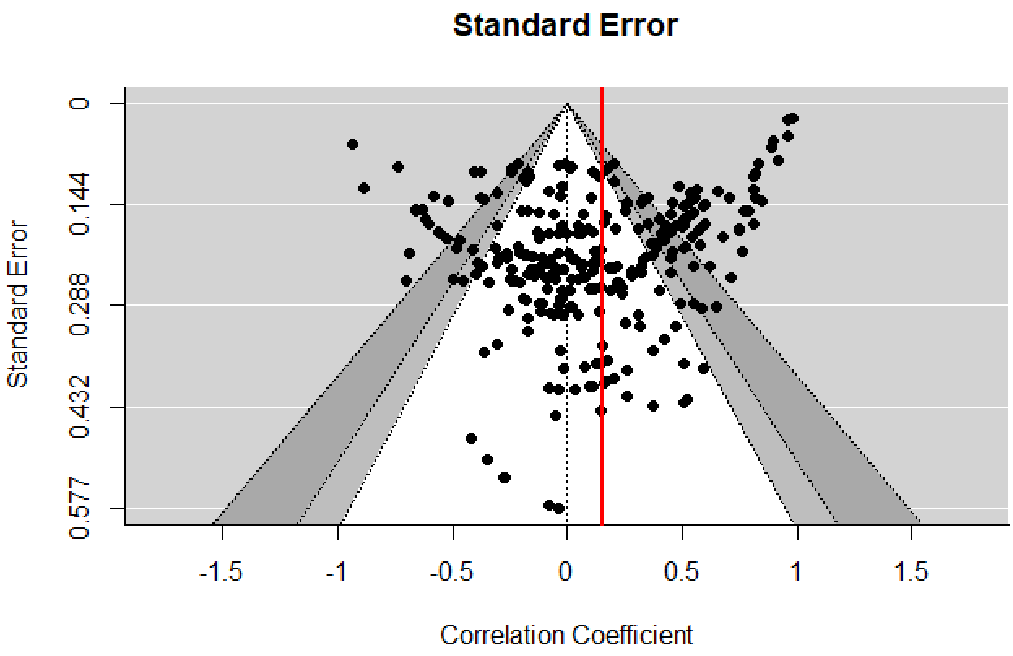

Figure S3. Funnel plot used to determine publication bias.

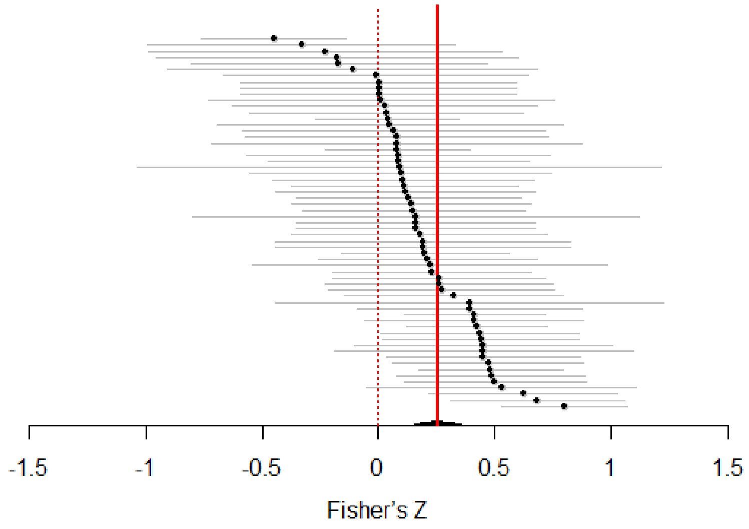

Figure S4. The 61 effect sizes estimate for β-SGDC.

Table S1 Information for 40 studies

ID	Species	Reference	SD	GD
**1**	*Peromyscus maniculatus*	Vellend 2003	Species richness	Excepted heterozygosity
**2**	*Trillium grandiflorum*	Vellend 2004	Evenness	Excepted heterozygosity
**2**	*Trillium grandiflorum*	Vellend 2004	Species richness	Excepted heterozygosity
**2**	*Trillium grandiflorum*	Vellend 2004	Evenness	Allelic richness
**2**	*Trillium grandiflorum*	Vellend 2004	Species richness	Allelic richness
**3**	*Ranunculus acris*	Odat et al. 2004	Species richness	Excepted heterozygosity
**3**	*Ranunculus acris*	Odat et al. 2004	Evenness	Excepted heterozygosity
**4**	*Gypsophila fastigiata*	Vellend and Geber 2005	Shannon-Weiner	Excepted heterozygosity
**5**	*Maianthemum bifolium*	Vellend and Geber 2005	Species richness	Excepted heterozygosity
**6**	*Fagus sylvatica*	Vellend and Geber 2005	Simpson	Excepted heterozygosity
**7**	*Picea abies*	Vellend and Geber 2005	Simpson	Excepted heterozygosity
**8**	*Oryza glaberrima/sativa*	Vellend and Geber 2005	Shannon-Weiner	Shannon-Weiner
**9**	*Centrolenella prosoblepon*	Vellend and Geber 2005	Species richness	Excepted heterozygosity
**10**	*Drupadia theda*	Cleary et al. 2006	Species richness	Allelic richness
**10**	*Drupadia theda*	Cleary et al. 2006	Species richness	Allelic richness
**11**	Total	Wehenkel et al. 2006	Species diversity	Genetic diversity
**11**	PGI	Wehenkel et al. 2006	Species diversity	Genetic diversity
**11**	HEK	Wehenkel et al. 2006	Species diversity	Genetic diversity
**11**	AAT	Wehenkel et al. 2006	Species diversity	Genetic diversity
**12**	*Banksia attenuata*	He et al. 2008	Species richness	Excepted heterozygosity
**12**	*Banksia attenuata*	He et al. 2008	Evenness	Allelic richness
**12**	*Banksia attenuata*	He et al. 2008	Species richness	Allelic richness

**12**	*Banksia attenuata*	He et al. 2008	Evenness	Excepted heterozygosity
**13**	*Carex curvula*	Puşcaş et al. 2008	Species richness	Excepted heterozygosity
**13**	*Carex curvula*	Puşcaş et al. 2008	Species richness	Excepted heterozygosity
**14**	*Antboxanthum odoratum*	Silvertown et al.2008	Species richness	Genetic diversity
**15**	*Carex sempervirens*	Yu et al. 2009	Species richness	Polymorphism
**15**	*Carex sempervirens*	Yu et al. 2009	Shannon	Polymorphism
**15**	*Carex sempervirens*	Yu et al. 2009	Evenness	Polymorphism
**15**	*Carex sempervirens*	Yu et al. 2009	Species richness	Genetic diversity
**15**	*Carex sempervirens*	Yu et al. 2009	Shannon	Genetic diversity
**15**	*Carex sempervirens*	Yu et al. 2009	Evenness	Genetic diversity
**16**	*Pecos gambusia*	Sei et al 2009	Species richness	Allelic richness
**17**	*Leptodiaptomus minutus*	Derry et al. 2009	Species richness	Excepted heterozygosity
**17**	*Leptodiaptomus minutus*	Derry et al. 2009	Evenness	Excepted heterozygosity
**18**	*Briza media*	Helm et al. 2009	Species richness	Polymorphism
**18**	*Briza media*	Helm et al. 2009	Species richness	Allelic richness
**18**	*Briza media*	Helm et al. 2009	Species richness	Genetic diversity
**19**	*Daviesia triflora*	He and Lamont 2010	Species richness	Allelic richness
**20**	*Plantago lanceolata*	Odat. et al 2010	Species richness	Gene diversity
**20**	*Plantago lanceolata*	Odat. et al 2010	Evenness	Gene diversity
**20**	*Plantago lanceolata*	Odat. et al 2010	Species richness	Gene diversity
**20**	*Plantago lanceolata*	Odat. et al 2010	Species richness	Gene diversity
**20**	*Plantago lanceolata*	Odat. et al 2010	Species richness	Gene diversity
**20**	*Plantago lanceolata*	Odat. et al 2010	Species richness	Gene diversity
**20**	*Plantago lanceolata*	Odat. et al 2010	Species richness	Gene diversity
**21**	*Crassostrea virginica*	Robinsonet al. 2010	Species richness	Nucleotide diversity
**21**	*Geukensia demissa*	Robinsonet al. 2010	Species richness	Nucleotide diversity

**21**	*Ilyanassa obsoleta*	Robinsonet al. 2010	Species richness	Nucleotide diversity
**21**	*Littoraria irrorata*	Robinsonet al. 2010	Species richness	Nucleotide diversity
**21**	*Melampus bidentatus*	Robinsonet al. 2010	Species richness	Nucleotide diversity
**21**	*Petrolisthes armatus*	Robinsonet al. 2010	Species richness	Nucleotide diversity
**21**	*Uca minax*	Robinsonet al. 2010	Species richness	Nucleotide diversity
**21**	*Uca pugnax*	Robinsonet al. 2010	Species richness	Nucleotide diversity
**22**	*Tenebrionid beetle*	Papadopoulou et al. 2011	Species richness	Nucleotide diversity
**22**	*Tenebrionid beetle*	Papadopoulou et al. 2011	Species richness	Nucleotide diversity
**22**	*Tenebrionid beetle*	Papadopoulou et al. 2011	Species richness	Nucleotide diversity
**22**	*Tenebrionid beetle*	Papadopoulou et al. 2011	Species richness	Nucleotide diversity
**22**	*Tenebrionid beetle*	Papadopoulou et al. 2011	Species richness	Nucleotide diversity
**22**	*Tenebrionid beetle*	Papadopoulou et al. 2011	Species richness	Nucleotide diversity
**22**	*Tenebrionid beetle*	Papadopoulou et al. 2011	Species richness	Nucleotide diversity
**22**	*Tenebrionid beetle*	Papadopoulou et al. 2011	Species richness	Nucleotide diversity
**22**	*Tenebrionid beetle*	Papadopoulou et al. 2011	Species richness	Nucleotide diversity
**22**	*Tenebrionid beetle*	Papadopoulou et al. 2011	Species richness	Nucleotide diversity
**22**	*Tenebrionid beetle*	Papadopoulou et al. 2011	Species richness	Nucleotide diversity
**22**	*Tenebrionid beetle*	Papadopoulou et al. 2011	Species richness	Nucleotide diversity
**22**	*Tenebrionid beetle*	Papadopoulou et al. 2011	Species richness	Nucleotide diversity
**22**	*Tenebrionid beetle*	Papadopoulou et al. 2011	Species richness	Nucleotide diversity
**22**	*Tenebrionid beetle*	Papadopoulou et al. 2011	Species richness	Nucleotide diversity
**22**	*Tenebrionid beetle*	Papadopoulou et al. 2011	Species richness	Nucleotide diversity
**22**	*Tenebrionid beetle*	Papadopoulou et al. 2011	Species richness	Nucleotide diversity
**22**	*Tenebrionid beetle*	Papadopoulou et al. 2011	Species richness	Nucleotide diversity
**22**	*Tenebrionid beetle*	Papadopoulou et al. 2011	Species richness	Nucleotide diversity
**22**	*Tenebrionid beetle*	Papadopoulou et al. 2011	Species richness	Nucleotide diversity
**22**	*Tenebrionid beetle*	Papadopoulou et al. 2011	Species richness	Nucleotide diversity

**22**	*Tenebrionid beetle*	Papadopoulou et al. 2011	Species richness	Nucleotide diversity
**22**	*Tenebrionid beetle*	Papadopoulou et al. 2011	Species richness	Nucleotide diversity
**22**	*Tenebrionid beetle*	Papadopoulou et al. 2011	Species richness	Nucleotide diversity
**23**	*Kerivoula papillosa*	Struebig et al. 2011	Species richness	Allelic richness
**23**	*Rhinolophus lepidus*	Struebig et al. 2011	Species richness	Allelic richness
**23**	*Rhinolophus lepidus*	Struebig et al. 2011	Species richness	Rarefied allelic richness
**24**	*Prosimulium neomacropyga*	Finn and Poff 2011	Species richness	Allelic richness
**25**	*Euptelea pleiospermum*	Wei et al 2012	Species richness	Allelic richness
**25**	*Euptelea pleiospermum*	Wei et al 2012	Species richness	Excepted heterozygosity
**25**	*Euptelea pleiospermum*	Wei et al 2012	Evenness	Allelic richness
**25**	*Euptelea pleiospermum*	Wei et al 2012	Evenness	Excepted heterozygosity
**26**	*Campostoma anomalum*	Blum et al. 2012	Species richness	Allelic richness
**26**	*Campostoma anomalum*	Blum et al. 2012	Species richness	Allelic richness
**26**	*Campostoma anomalum*	Blum et al. 2012	Evenness	Excepted heterozygosity
**26**	*Campostoma anomalum*	Blum et al. 2012	Evenness	Excepted heterozygosity
**27**	*Andropogon gerardii*	Avolio et al 2012	Genotypic richness	Allelic richness
**27**	*Andropogon gerardii*	Avolio et al 2012	Genotypic richness	Allelic richness
**28**	*Androsace obtusifolia*	Taberlet et al. 2013	Species richness	Genetic diversity
**28**	*Arabis alpina*	Taberlet et al. 2013	Species richness	Genetic diversity
**28**	*Campanula barbata*	Taberlet et al. 2013	Species richness	Genetic diversity
**28**	*Carex firma*	Taberlet et al. 2013	Species richness	Genetic diversity
**28**	*Carex sempervirens*	Taberlet et al. 2013	Species richness	Genetic diversity
**28**	*Cerastium uniflorum*	Taberlet et al. 2013	Species richness	Genetic diversity
**28**	*Cirsium spinosissimum*	Taberlet et al. 2013	Species richness	Genetic diversity
**28**	*Dryas octopetala*	Taberlet et al. 2013	Species richness	Genetic diversity
**28**	*Gentiana nivalis*	Taberlet et al. 2013	Species richness	Genetic diversity

**28**	*Geum montanum*	Taberlet et al. 2013	Species richness	Genetic diversity
**28**	*Geum reptans*	Taberlet et al. 2013	Species richness	Genetic diversity
**28**	*Gypsophila repens*	Taberlet et al. 2013	Species richness	Genetic diversity
**28**	*Hedysarum hedysaroides*	Taberlet et al. 2013	Species richness	Genetic diversity
**28**	*Hornungia alpina*	Taberlet et al. 2013	Species richness	Genetic diversity
**28**	*Hypochaeris uniflora*	Taberlet et al. 2013	Species richness	Genetic diversity
**28**	*Juncus trifidus*	Taberlet et al. 2013	Species richness	Genetic diversity
**28**	*Ligusticum mutellinoides*	Taberlet et al. 2013	Species richness	Genetic diversity
**28**	*Loiseleuria procumbens*	Taberlet et al. 2013	Species richness	Genetic diversity
**28**	*Luzula alpinopilosa*	Taberlet et al. 2013	Species richness	Genetic diversity
**28**	*Peucedanum ostruthium*	Taberlet et al. 2013	Species richness	Genetic diversity
**28**	*Phyteuma betonicifolium*	Taberlet et al. 2013	Species richness	Genetic diversity
**28**	*Phyteuma hemisphaericum*	Taberlet et al. 2013	Species richness	Genetic diversity
**28**	*Ranunculus alpestris*	Taberlet et al. 2013	Species richness	Genetic diversity
**28**	*Rhododendron ferrugineum*	Taberlet et al. 2013	Species richness	Genetic diversity
**28**	*Saxifraga stellaris*	Taberlet et al. 2013	Species richness	Genetic diversity
**28**	*Sesleria caerulea*	Taberlet et al. 2013	Species richness	Genetic diversity
**28**	*Trifolium alpinum*	Taberlet et al. 2013	Species richness	Genetic diversity
**29**	*Arabis alpina*	Taberlet et al. 2013	Species richness	Genetic diversity
**29**	*Campanula alpina*	Taberlet et al. 2013	Species richness	Genetic diversity
**29**	*Campanula serrata*	Taberlet et al. 2013	Species richness	Genetic diversity
**29**	*Carex sempervirens*	Taberlet et al. 2013	Species richness	Genetic diversity
**29**	*Dryas octopetala*	Taberlet et al. 2013	Species richness	Genetic diversity
**29**	*Festuca carpathica*	Taberlet et al. 2013	Species richness	Genetic diversity
**29**	*Festuca supina*	Taberlet et al. 2013	Species richness	Genetic diversity
**29**	*Festuca versicolor*	Taberlet et al. 2013	Species richness	Genetic diversity

**29**	*Gentiana nivalis*	Taberlet et al. 2013	Species richness	Genetic diversity
**29**	*Geum montanum*	Taberlet et al. 2013	Species richness	Genetic diversity
**29**	*Geum reptans*	Taberlet et al. 2013	Species richness	Genetic diversity
**29**	*Hedysarum hedysaroides*	Taberlet et al. 2013	Species richness	Genetic diversity
**29**	*Hypochaeris uniflora*	Taberlet et al. 2013	Species richness	Genetic diversity
**29**	*Juncus trifidus*	Taberlet et al. 2013	Species richness	Genetic diversity
**29**	*Loiseleuria procumbens*	Taberlet et al. 2013	Species richness	Genetic diversity
**29**	*Luzula alpinopilosa*	Taberlet et al. 2013	Species richness	Genetic diversity
**29**	*Phyteuma confusum*	Taberlet et al. 2013	Species richness	Genetic diversity
**29**	*Primula minima*	Taberlet et al. 2013	Species richness	Genetic diversity
**29**	*Rhododendron myrthifolium*	Taberlet et al. 2013	Species richness	Genetic diversity
**29**	*Saxifraga stellaris*	Taberlet et al. 2013	Species richness	Genetic diversity
**29**	*Saxifraga wahlenbergii*	Taberlet et al. 2013	Species richness	Genetic diversity
**29**	*Sempervivum montanum*	Taberlet et al. 2013	Species richness	Genetic diversity
**29**	*Soldanella pusilla*	Taberlet et al. 2013	Species richness	Genetic diversity
**29**	*Veronica baumgartenii*	Taberlet et al. 2013	Species richness	Genetic diversity
**30**	*Drepanotrema depressissimum*	Lamy et al. 2013	Species richness	Allelic richness
**30**	*Drepanotrema depressissimum*	Lamy et al. 2013	Species richness	Genetic diversity
**30**	*Aplexa marmorata*	Lamy et al. 2013	Species richness	Allelic richness
**30**	*Aplexa marmorata*	Lamy et al. 2013	Species richness	Genetic diversity
**31**	*Andropogon gerardii*	Avolio and Smith 2013	Species richness	Nucleotide diversity
**31**	*Andropogon gerardii*	Avolio and Smith 2013	Evenness	Nucleotide diversity
**31**	*Andropogon gerardii*	Avolio and Smith 2013	Shannon	Nucleotide diversity
**31**	*Andropogon gerardii*	Avolio and Smith 2013	Species richness	Genomic dissimilarity
**31**	*Andropogon gerardii*	Avolio and Smith 2013	Evenness	Genomic dissimilarity
**31**	*Andropogon gerardii*	Avolio and Smith 2013	Shannon	Genomic dissimilarity

**32**	*Aribeus jamaicaensis*	Vellend et al. 2014	Species richness	Excepted heterozygosity
**33**	*Brachyphylla cavernarum*	Vellend et al. 2014	Species richness	Excepted heterozygosity
**34**	*Carex rariflora*	Vellend et al. 2014	Species richness	Excepted heterozygosity
**35**	*Anthyllis vulneraria*	Vellend et al. 2014	Species richness	Excepted heterozygosity
**36**	*Primula elatior*	Vellend et al. 2014	Species richness	Excepted heterozygosity
**37**	*Gammarus spp.*	Vellend et al. 2014	Species richness	Excepted heterozygosity
**38**	*Gambusia nobilis*	Vellend et al. 2014	Species richness	Excepted heterozygosity
**39**	*Gammarus spp.*	Vellend et al. 2014	Species richness	Excepted heterozygosity
**40**	*Gambusia nobilis*	Vellend et al. 2014	Species richness	Excepted heterozygosity
**41**	*Saponaria bellidifolia*	Csergő et al. 2014	Species richness	Band richness
**41**	*Saponaria bellidifolia*	Csergő et al. 2014	Species richness	Excepted heterozygosity
**42**	*Potamogeton pectinatus*	Han et al. 2014	Species richness	Excepted heterozygosity
**42**	*Potamogeton pectinatus*	Han et al. 2014	Evenness	Excepted heterozygosity
**42**	*Potamogeton pectinatus*	Han et al. 2014	Species richness	Shannon
**42**	*Potamogeton pectinatus*	Han et al. 2014	Evenness	Shannon
**43**	*Picea chihuahuana*	Sergio et al 2014	Species richness	Allelic richness
**43**	*Picea chihuahuana*	Sergio et al 2014	Simpson	Allelic richness
**43**	*Picea chihuahuana*	Sergio et al 2014	Evenness	Allelic richness
**43**	*Pinus strobiformis*	Sergio et al 2014	Species richness	Allelic richness
**43**	*Pinus strobiformis*	Sergio et al 2014	Simpson	Allelic richness
**43**	*Pinus strobiformis*	Sergio et al 2014	Evenness	Allelic richness
**43**	*Populus tremuloides*	Sergio et al 2014	Species richness	Allelic richness
**43**	*Populus tremuloides*	Sergio et al 2014	Simpson	Allelic richness
**43**	*Populus tremuloides*	Sergio et al 2014	Evenness	Allelic richness
**43**	*Pseudotsuga menziesii*	Sergio et al 2014	Species richness	Allelic richness
**43**	*Pseudotsuga menziesii*	Sergio et al 2014	Simpson	Allelic richness

**43**	*Pseudotsuga menziesii*	Sergio et al 2014	Evenness	Allelic richness
**44**	*Focal species*	Frey et al 2016	Species richness	Shannon
**44**	*Focal species*	Frey et al 2016	Species richness	Rarity index
**44**	*Focal species*	Frey et al 2016	Evenness	Shannon
**44**	*Focal species*	Frey et al 2016	Evenness	Rarity index
**45**	*Beilschmiedia roxburghiana*	Xu et al 2016	Rarefied tree richness	Rarefied number of alleles
**45**	*Beilschmiedia roxburghiana*	Xu et al 2016	SW_GD	SW_TD
**45**	*Beilschmiedia roxburghiana*	Xu et al 2016	Species richness	Allelic richness
**45**	*Beilschmiedia roxburghiana*	Xu et al 2016	PC1_GD	PC1_GD
**46**	*Euptelea pleiospermum*	Wei et al 2016	Species richness	Allelic richness
**46**	*Euptelea pleiospermum*	Wei et al 2016	Species richness	Excepted heterozygosity
**46**	*Euptelea pleiospermum*	Wei et al 2016	Evenness	Allelic richness
**46**	*Euptelea pleiospermum*	Wei et al 2016	Evenness	Excepted heterozygosity
**46**	*Euptelea pleiospermum*	Wei et al 2016	Species richness	Allelic richness
**46**	*Euptelea pleiospermum*	Wei et al 2016	Species richness	Excepted heterozygosity
**46**	*Euptelea pleiospermum*	Wei et al 2016	Evenness	Allelic richness
**46**	*Euptelea pleiospermum*	Wei et al 2016	Evenness	Excepted heterozygosity
**47**	*C. gayana*	Bertin et al .2017	Species richness	Nei's gene diversity
**47**	*C. gayana*	Bertin et al .2017	Species richness	Nei's gene diversity
**47**	*C. gayana*	Bertin et al .2017	Species richness	FST
**47**	*P. clandestina*	Bertin et al .2017	Species richness	Nei's gene diversity
**47**	*P. clandestina*	Bertin et al .2017	Species richness	Nei's gene diversity
**47**	*P. clandestina*	Bertin et al .2017	Species richness	FST
**47**	*H. fossamancinii*	Bertin et al .2017	Species richness	Nei's gene diversity
**47**	*H. fossamancinii*	Bertin et al .2017	Species richness	Nei's gene diversity
**47**	*H. fossamancinii*	Bertin et al .2017	Species richness	FST

**47**	*A. peruvianus*	Bertin et al .2017	Species richness	Nei's gene diversity
**47**	*A. peruvianus*	Bertin et al .2017	Species richness	Nei's gene diversity
**47**	*A. peruvianus*	Bertin et al .2017	Species richness	FST
**47**	*Austrelmis sp*	Bertin et al .2017	Species richness	Nei's gene diversity
**47**	*Austrelmis sp*	Bertin et al .2017	Species richness	Nei's gene diversity
**47**	*Austrelmis sp*	Bertin et al .2017	Species richness	FST
**47**	Mean	Bertin et al .2017	Species richness	Nei's gene diversity
**47**	Mean	Bertin et al .2017	Species richness	Nei's gene diversity
**47**	Mean	Bertin et al .2017	Species richness	FST
**48**	*Hydropsyche orientallis*	Watanabe et al 2017	Taxon richness (Str)	Nonneutral
**48**	*Hydropsyche orientallis*	Watanabe et al 2017	Relative abundance	Nonneutral
**48**	*Hydropsyche orientallis*	Watanabe et al 2017	Taxon richness (S)	Nonneutral
**48**	*Hydropsyche orientallis*	Watanabe et al 2017	Relative abundance	Nonneutral
**48**	*Hydropsyche orientallis*	Watanabe et al 2017	Taxon richness (Str)	Neutral
**48**	*Hydropsyche orientallis*	Watanabe et al 2017	Relative abundance	Neutral
**48**	*Hydropsyche orientallis*	Watanabe et al 2017	Taxon richness (S)	Neutral
**48**	*Hydropsyche orientallis*	Watanabe et al 2017	Relative abundance	Neutral
**48**	*Stenopsyche marmorata*	Watanabe et al 2017	Taxon richness (Str)	Nonneutral
**48**	*Stenopsyche marmorata*	Watanabe et al 2017	Relative abundance	Nonneutral
**48**	*Stenopsyche marmorata*	Watanabe et al 2017	Taxon richness (S)	Nonneutral
**48**	*Stenopsyche marmorata*	Watanabe et al 2017	Relative abundance	Nonneutral
**48**	*Stenopsyche marmorata*	Watanabe et al 2017	Taxon richness (Str)	Neutral
**48**	*Stenopsyche marmorata*	Watanabe et al 2017	Relative abundance	Neutral
**48**	*Stenopsyche marmorata*	Watanabe et al 2017	Taxon richness (S)	Neutral
**48**	*Stenopsyche marmorata*	Watanabe et al 2017	Relative abundance	Neutral
**48**	*Hydropsyche albicephala*	Watanabe et al 2017	Taxon richness (Str)	Nonneutral

**48**	*Hydropsyche albicephala*	Watanabe et al 2017	Relative abundance	Nonneutral
**48**	*Hydropsyche albicephala*	Watanabe et al 2017	Taxon richness (S)	Nonneutral
**48**	*Hydropsyche albicephala*	Watanabe et al 2017	Relative abundance	Nonneutral
**48**	*Hydropsyche albicephala*	Watanabe et al 2017	Taxon richness (Str)	Neutral
**48**	*Hydropsyche albicephala*	Watanabe et al 2017	Relative abundance	Neutral
**48**	*Hydropsyche albicephala*	Watanabe et al 2017	Taxon richness (S)	Neutral
**48**	*Hydropsyche albicephala*	Watanabe et al 2017	Relative abundance	Neutral
**48**	*Ephemera japonica*	Watanabe et al 2017	Taxon richness (Str)	Nonneutral
**48**	*Ephemera japonica*	Watanabe et al 2017	Relative abundance	Nonneutral
**48**	*Ephemera japonica*	Watanabe et al 2017	Taxon richness (S)	Nonneutral
**48**	*Ephemera japonica*	Watanabe et al 2017	Relative abundance	Nonneutral
**48**	*Ephemera japonica*	Watanabe et al 2017	Taxon richness (Str)	Neutral
**48**	*Ephemera japonica*	Watanabe et al 2017	Relative abundance	Neutral
**48**	*Ephemera japonica*	Watanabe et al 2017	Taxon richness (S)	Neutral
**48**	*Ephemera japonica*	Watanabe et al 2017	Relative abundance	Neutral
**49**	*Primula veris*	Reisch et al 2018	Simpson	Nei's gene diversity
**49**	*Primula veris*	Reisch et al 2018	Specialists	Nei's gene diversity
**49**	*Dianthus carthusianorum*	Reisch et al 2018	Simpson	Nei's gene diversity
**49**	*Dianthus carthusianorum*	Reisch et al 2018	Specialists	Nei's gene diversity
**49**	*Medicago falcata*	Reisch et al 2018	Simpson	Nei's gene diversity
**49**	*Medicago falcata*	Reisch et al 2018	Specialists	Nei's gene diversity
**49**	*Polygala comosa*	Reisch et al 2018	Simpson	Nei's gene diversity
**49**	*Polygala comosa*	Reisch et al 2018	Specialists	Nei's gene diversity
**49**	*Salvia pratensis*	Reisch et al 2018	Simpson	Nei's gene diversity
**49**	*Salvia pratensis*	Reisch et al 2018	Specialists	Nei's gene diversity
**49**	Mean	Reisch et al 2018	Simpson	Nei's gene diversity

**49**	Mean	Reisch et al 2018	Specialists	Nei's gene diversity
**50**	*Carex gayana*	Pfeiffer et al 2018	Species richness	DS1
**50**	*Carex gayana*	Pfeiffer et al 2018	Species richness	DS1
**50**	*Carex gayana*	Pfeiffer et al 2018	Evenness	DS1
**50**	*Carex gayana*	Pfeiffer et al 2018	Evenness	DS1
**50**	*Carex gayana*	Pfeiffer et al 2018	Species richness	DS2
**50**	*Carex gayana*	Pfeiffer et al 2018	Species richness	DS2
**50**	*Carex gayana*	Pfeiffer et al 2018	Evenness	DS2
**50**	*Carex gayana*	Pfeiffer et al 2018	Evenness	DS2
**50**	*Carex gayana*	Pfeiffer et al 2018	Species richness	DS3
**50**	*Carex gayana*	Pfeiffer et al 2018	Species richness	DS3
**50**	*Carex gayana*	Pfeiffer et al 2018	Evenness	DS3
**50**	*Carex gayana*	Pfeiffer et al 2018	Evenness	DS3
**50**	*Carex gayana*	Pfeiffer et al 2018	Species richness	DS4
**50**	*Carex gayana*	Pfeiffer et al 2018	Species richness	DS4
**50**	*Carex gayana*	Pfeiffer et al 2018	Evenness	DS4
**50**	*Carex gayana*	Pfeiffer et al 2018	Evenness	DS4
**50**	*Carex gayana*	Pfeiffer et al 2018	Species richness	DS5
**50**	*Carex gayana*	Pfeiffer et al 2018	Species richness	DS5
**50**	*Carex gayana*	Pfeiffer et al 2018	Evenness	DS5
**50**	*Carex gayana*	Pfeiffer et al 2018	Evenness	DS5
**51**	*Rana temporaria*	Marchesini et al., 2018	Species richness	Allelic richness
**51**	*Rana temporaria*	Marchesini et al., 2018	Species richness	Allelic richness
**52**	*Potamogeton wrghtii*	Henry 2019	Species richness	Excepted heterozygosity
**52**	*Potamogeton wrghtii*	Henry 2019	Species richness	Allelic richness
**52**	*Potamogeton wrghtii*	Henry 2019	Evenness	Excepted heterozygosity

**52**	*Potamogeton wrghtii*	Henry 2019	Evenness	Allelic richness
**52**	*Myriophllum spicatum*	Henry 2019	Species richness	Excepted heterozygosity
**52**	*Myriophllum spicatum*	Henry 2019	Species richness	Allelic richness
**52**	*Myriophllum spicatum*	Henry 2019	Evenness	Excepted heterozygosity
**52**	*Myriophllum spicatum*	Henry 2019	Evenness	Allelic richness
**53**	*Artibeus planirostris*	Lino et al 2020	Species richness	Allelic richness
**53**	*Artibeus planirostris*	Lino et al 2020	Species richness	Excepted heterozygosity
**53**	*Artibeus planirostris*	Lino et al 2020	Evenness	Allelic richness
**53**	*Artibeus planirostris*	Lino et al 2020	Evenness	Excepted heterozygosity
**53**	*Carollia perspicillata*	Lino et al 2020	Species richness	Allelic richness
**53**	*Carollia perspicillata*	Lino et al 2020	Species richness	Excepted heterozygosity
**53**	*Carollia perspicillata*	Lino et al 2020	Evenness	Allelic richness
**53**	*Carollia perspicillata*	Lino et al 2020	Evenness	Excepted heterozygosity

Table S2 Interaction of two categorical variables on effects of SGDC, *p*-value <0.05 are shown in the table.

	Y	N	Plant	Animal	Wetland	Island	Grassland	Forest	RAPD	mtDNA	Microsatellite	Allozyme	AFLP
Y			0.2850	0.1836	0.3104	0.4602		0.2305	0.7923	0.3191	0.2376		0.3157
N				0.2602							0.2967		
Plant	0.2850												0.1131
Animal	0.1836	0.2602				0.4697				0.3606	0.3128		
Wetland	0.3104		0.3936										
Island	0.4602			0.4697						0.0411			
Grassland													
Forest	0.2305								0.0343				
RAPD	0.7923							0.0343					
mtDNA	0.3191			0.3606	0.0004	0.0411							
Microsatellite	0.2376	0.2967		0.3128		0.4183							
Allozyme													
AFLP	0.3157		0.1131		0.387								

Text A1. SGDC for studies included in the meta-analysis.

Avolio, M. L., J. M. Beaulieu, E. Y. Y. Lo, and M. D. Smith. 2012. Measuring genetic diversity in ecological studies. Plant Ecology, 213, 1105-1115.

Avolio, M. L., M. D. Smith, and R. Michalet. 2013. Correlations between genetic and species diversity: effects of resource quantity and heterogeneity. *Journal of Vegetation Science*, *24*,1185-1194.

Bertin, A., N. Gouin, A. Baumel, E. Gianoli, J. Serratosa, R. Osorio, and S. Manel. 2017. Genetic variation of loci potentially under selection confounds species-genetic diversity correlations in a fragmented habitat. *Molecular Ecology*, *26*, 431-443.

Blum, M. J., M. J. Bagley, D. M. Walters, S. A. Jackson, F. B. Daniel, D. J. Chaloud, and B. S. Cade. 2012. Genetic diversity and species diversity of stream fishes covary across a land-use gradient. *Oecologia*, *168*, 83-95.

Cleary DFR, Fauvelot C, Genner MJ, Menken SBJ, Mooers AØ. 2006. Parallel responses of species and diversity to El Niño Southern Oscillation-induced environmental destruction. *Ecology Letters*, *9*, 304–310.

Derry, A. M., S. E. Arnott, J. A. Shead, P. D. N. Hebert, and P. T. Boag. 2010. Ecological linkages between community and genetic diversity in zooplankton among boreal shield lakes. *Ecology*, *90*, 2275-2286.

Finn, D. S., and N. L. Poff. 2011. Examining spatial concordance of genetic and species diversity patterns to evaluate the role of dispersal limitation in structuring headwater metacommunities. *Journal of the North American Benthological Society* 30:273-283.

Frey, D., N. Arrigo, G. Granereau, A. Sarr, F. Felber, and G. Kozlowski. 2016. Parallel declines in species and genetic diversity driven by anthropogenic disturbance: a multispecies approach in a French Atlantic dune system. *Evolutionary Applications*, *9*, 479-488.

Han, Q. X., G. X. Wang, W. Li, and F. Liu. 2014. Genetic diversity of Potamogeton pectinatus L. in relation to species diversity in a pair of lakes of contrasting trophic levels. *Biochemical Systematics and Ecology* 57:60-66.

He, T. H., and B. B. Lamont. 2010. Species versus genotypic diversity of a nitrogen-fixing plant functional group in a metacommunity. *Population Ecology*, *52*, 337-345.

He, T. H., B. B. Lamont, S. L. Krauss, N. J. Enright, and B. P. Miller. 2008. Covariation between intraspecific genetic diversity and species diversity within a plant functional group. *Journal of Ecology*, *96*, 956-961.

Helm A, Oja T, Saar L, Takkis K, Talve T, Pärtel M. 2009. Human influence lowers plant genetic diversity in communities with extinction debt. *Journal of Ecology*, *97*, 1329–1336.

Lamy, T., P. Jarne, F. Laroche, J. P. Pointier, G. Huth, A. Segard, and P. David. 2013. Variation in habitat connectivity generates positive correlations between species and genetic diversity in a metacommunity. *Molecular Ecology*, *22*, 4445-4456.

Lino, A., E. Ferreira, C. Fonseca, E. Fischer, and M. J. R. Pereira. 2021. Species-genetic diversity correlation in phyllostomid bats of the Bodoquena plateau, Brazil. Biodiversity and Conservation 30:403-429.

Henry, N. K. 2019. Study on relationship between species-genetic diversity of two dominant submerged plants in honghu, lake. Chinese acsdemy of scieneces.

Marchesini A , Vernesi C , Battisti A. , Ficetola, G. F.. 2018. Deciphering the drivers of negative species–genetic diversity correlation in Alpine amphibians. Molecular Ecology, 27.

Odat, N., F. H. Hellwig, G. Jetschke, and M. Fischer. 2010. On the relationship between plant species diversity and genetic diversity of Plantago lanceolata (Plantaginaceae) within and between grassland communities. *Journal of Plant Ecology*, *3*, 41-48.

Odat, N., G. Jetschke, and F. H. Hellwig. 2004. Genetic diversity of Ranunculus acris L. (Ranunculaceae) populations in relation to species diversity and habitat type in grassland communities. *Molecular Ecology*, *13*, 1251-1257.

Papadopoulou, A., I. Anastasiou, F. Spagopoulou, M. Stalimerou, S. Terzopoulou, A. Legakis, and A. P. Vogler. 2011. Testing the species--genetic diversity correlation in the Aegean archipelago: toward a haplotype-based macroecology? *Am Nat*, *178*, 241-255.

Pfeiffer, V. W., B. M. Ford, J. Housset, A. McCombs, J. L. Blanco-Pastor, N. Gouin, S. Manel, and A. Bertin. 2018. Partitioning genetic and species diversity refines our understanding of species-genetic diversity relationships. *Ecol Evol*, *8*, 12351-12364.

Puşcaş, M., P. Taberlet, and P. Choler. 2008. No positive correlation between species and genetic diversity in European alpine grasslands dominated byCarex curvula. *Diversity and Distributions*, *14*, 852-861.

Reisch, C., and C. Schmid. 2019. Species and genetic diversity are not congruent in fragmented dry grasslands. *Ecol Evol*, *9*, 664-671.

Robinson, J. D., E. Diaz-Ferguson, M. F. Poelchau, S. Pennings, T. D. Bishop, and J. Wares. 2010. Multiscale Diversity in the Marshes of the Georgia Coastal Ecosystems LTER. Estuaries and Coasts, 33, 865-877.

Silvertown, J., P. M. Biss, and J. Freeland. 2009. Community genetics: resource addition has opposing effects on genetic and species diversity in a 150-year experiment. *Ecology Letters*, *12*, 165-170.

Simental-Rodriguez, S. L., C. Z. Quinones-Perez, D. Moya, E. Hernandez-Tecles, C. A. Lopez-Sanchez, and C. Wehenkel. 2014. The Relationship between Species Diversity and Genetic Structure in the Rare Picea chihuahuana Tree Species Community, Mexico. *Plos One*, *9*.

Struebig MJ, Kingston T, Petit EJ, Le Comber SC, Zubaid A, Mohd-Adnan A, Rossiter SJ. 2011 Parallel declines in species and genetic diversity in tropical forest fragments. *Ecology Letters*, *14*, 582–590.

Taberlet, P., N. E. Zimmermann, T. Englisch, A. Tribsch, R. Holderegger, N. Alvarez, H. Niklfeld, G. Coldea, Z. Mirek, A. Moilanen, W. Ahlmer, P. A. Marsan, E. Bona, M. Bovio, P. Choler, E. Cieslak, L. Colli, V. Cristea, J. P. Dalmas, B. Frajman, L. Garraud, M. Gaudeul, L. Gielly, W. Gutermann, N. Jogan, A. A. Kagalo, G. Korbecka, P. Kupfer, B. Lequette, D. R. Letz, S. Manel, G. Mansion, K. Marhold, F. Martini, R. Negrini, F. Nino, O. Paun, M. Pellecchia, G. Perico, H. Piekos-Mirkowa,

F. Prosser, M. Puscas, M. Ronikier, M. Scheuerer, G. M. Schneeweiss, P. Schonswetter, L. Schratt-Ehrendorfer, F. Schupfer, A. Selvaggi, K. Steinmann, C. Thiel-Egenter, M. van Loo, M. Winkler, T. Wohlgemuth, T. Wraber, F. Gugerli, C. IntraBioDiv, and M. Vellend. 2012. Genetic diversity in widespread species is not congruent with species richness in alpine plant communities. *Ecol Lett*, *15*, 1439-1448.

Vellend, M. 2003. Island biogeography of genes and species. Am Nat 162:358-365.

Vellend, M. 2004. Parallel effects of land-use history on species diversity and genetic diversity of forest herbs. *Ecology*, *85*, 3043-3055.

Vellend, M., and M. A. Geber. 2005. Connections between species diversity and genetic diversity. *Ecology Letters* 8:767-781.

Watanabe, K., and M. T. Monaghan. 2017. Comparative tests of the species-genetic diversity correlation at neutral and nonneutral loci in four species of stream insect. *Evolution*, *71*, 1755-1764.

Wehenkel C, Bergmann F, Hans-Rolf G. 2006. Is there a trade-off between species diversity and genetic diversity in forest tree communities? Plant Ecology, 185, 151–161

Wei, X., D. Bao, H. Meng, and M. Jiang. 2017. Pattern and drivers of species-genetic diversity correlation in natural forest tree communities across a biodiversity hotspot. *Journal of Plant Ecology*.

Wei, X., and M. Jiang. 2012. Contrasting relationships between species diversity and genetic diversity in natural and disturbed forest tree communities. *New Phytol*, *193*, 779-786.

Xu, W., L. Liu, T. He, M. Cao, L. Sha, Y. Hu, Q. Li, and J. Li. 2016. Soil properties drive a negative correlation between species diversity and genetic diversity in a tropical seasonal rainforest. *Sci Rep*, *6*, 20652.

Yu, F. H., B. O. Krusi, J. J. Schneller, M. Schutz, M. Tang, and O. Wildi. 2009. Positive correlation between vegetation dissimilarity and genetic differentiation of Carex sempervirens. *Flora* 204:651-657.
